# Socio-demographic and health service factors associated with antibiotic dispensing in older Australian adults

**DOI:** 10.1371/journal.pone.0221480

**Published:** 2019-08-29

**Authors:** Yingxi Chen, Martyn D. Kirk, Rhonda Stuart, Allen C. Cheng, Sallie-Anne Pearson, Andrew Hayen, Emily Banks, Bette Liu

**Affiliations:** 1 Research School of Population Health, Australian National University, Canberra, Australia; 2 School of Public Health and Community Medicine, University of New South Wales, Sydney, Australia; 3 Infection Prevention & Epidemiology, Monash Health, Melbourne, Australia; 4 National Centre for Antimicrobial Stewardship (NCAS), Monash University, Melbourne, Australia; 5 Infection Prevention & Epidemiology Unit, Alfred Health; School of Public Health and Preventive Medicine, Monash University, Melbourne, Australia; 6 Centre for Big Data Research in Health, University of New South Wales, Sydney, Australia; 7 Australian Centre for Public and Population Health Research, University of Technology Sydney, Sydney, Australia; University of South Australia, AUSTRALIA

## Abstract

**Background:**

Widespread use of antibiotics has led to the development of antibiotic resistance. However, there are limited data describing antibiotic use in the community setting, and examining factors associated with greater use. Our study aimed to quantify antibiotic dispensing in older adults in the community according to socio-demographics and health services use.

**Methods:**

Prospective analysis of a population-based cohort study of 239,981 adults aged ≥45 years in Australia (the Sax Institute’s 45 and Up Study). Data on socio-demographics and health from a questionnaire, were linked to 2015 antibiotic dispensing data from the Pharmaceutical Benefits Scheme (PBS), as well as other administrative health databases. We estimated the Defined Daily Dose (DDD) of systemic antibiotics dispensed, defined by an Anatomic Therapeutic Classification code beginning with J01, in 2015. We also conducted Poisson regression with robust standard errors to identify factors associated with antibiotic dispensing.

**Results:**

Overall, 49.3% of 45 and Up Study participants had at least one systemic antibiotic dispensed in 2015 with a total of 392,856 prescriptions dispensed and an average of 36.5 DDDs/1000-persons/day in the study population. The quantity of antibiotics dispensed increased with increasing age (25.6 DDDs/1000/day in <60 years old versus 50.4 DDDs/1000/day in 80+ year old) and was higher comparing women to men (39.9 versus 32.4 DDDs/1000/day). Of factors examined, the greatest dispensing of antibiotics was among those who had been resident in an aged care facility and those with >15 general practitioner consultations in the last year (80.5 and 88.3 DDDs/1000/day, respectively). These factors remained strongly associated with greater antibiotic dispensing after adjusting for age, sex, education, income, area of residence and co-morbidities.

**Conclusions:**

Residence in aged care facilities and high GP visits are associated with greater antibiotic dispensing. This study provides important evidence regarding high use groups for antimicrobial stewardship.

## Introduction

Antibiotics have been instrumental in reducing illness and death from infectious diseases. However, widespread use has led to the development of antibiotic resistance [1]. Antibiotic use in Australia is high. In 2015, 30 million antibiotic prescriptions were dispensed and 45% of the Australian population had at least one prescription for antibiotics [2]. Australia is ranked eighth among Organisation for Economic Co-operation and Development (OECD) countries with comparable data on antibiotic use [2].

While there are aggregated data on antibiotic use nationally [2] and in various institutional settings such as hospitals and long-term care facilities [3–5] there are limited data describing and quantifying antibiotic use in the community and examining the socio-demographic and health service factors associated with the greatest use. Knowledge regarding what factors are associated with greater antibiotic use in the community would enable research that focuses on high use groups, and potentially facilitate targeting of interventions to reduce or improve antibiotic use in such groups.

Hence the aim of this study was to quantify antibiotic dispensing in a cohort of community-based older Australians, and to identify socio-demographic and health service factors associated with greater antibiotic dispensing. Such descriptive data would increase our understanding of factors contributing to high antibiotic use in Australia and the potential to improve antibiotic stewardship in the community setting.

## Materials and methods

All participants provided written informed consent at the time of recruitment. The conduct of the 45 and Up Study was approved by UNSW Human Research Ethics Committee (HREC). This specific study was approved by the NSW Population Health Research Ethics Committee (2010/12/292)

### Data sources, study population and linkage

We used data from a large ongoing community-based population cohort study of adults aged 45 years and over—The Sax Institute’s 45 and Up Study. The study recruited 267000 adults in New South Wales (NSW), the most populous state in Australia, between 2006 and 2009. Participants were randomly sampled from the Department of Human Services (DHS, formerly Medicare Australia) enrolment database. The study recruited approximately 10% of the population in the target age range in NSW. At recruitment, participants completed a questionnaire [6], which included socio-demographic, health and lifestyle information [7]. Study participants agreed to be followed up by additional surveys and through linkage to their health records. A detailed description of the cohort and recruitment methods has been published [7].

For this study, we used individual participant data linked to: the Pharmaceutical Benefit Scheme (PBS); the Medicare Benefit Schedule (MBS); the NSW Admitted Patient Data Collection (APDC); and the NSW Register of Births, Deaths, and Marriages (RBDM). The PBS and MBS data were linked by the Sax Institute using a unique identifier provided by the DHS and the APDC and death data were linked by the NSW Centre for Health Record Linkage (CHeReL) using probabilistic linkage. All linkages were conducted independent of the study investigators and the CHeReL report false positive and false negative linkages of <0.5% and <0.1%, respectively [8].

The PBS records subsidised dispensed prescription drugs under the Pharmaceutical Benefits Scheme [9]. Information includes the pharmaceutical product, the Anatomical Therapeutic Chemical (ATC) classification code, strength, quantity and date of dispensing. Since July 2012 all claims for dispensed PBS medications were recorded on this dataset. The MBS records subsidised services under Australia’s publicly funded universal health insurance scheme, Medicare. Information includes all services, diagnostic procedures and tests that are listed under the MBS and reimbursed via Medicare. Subsidised health services and the date of service is also recorded.

The APDC documents all patient admissions to NSW hospitals. Information includes the principal diagnosis, up to 54 additional diagnoses contributing to the admission, procedures, and dates. The RBDM includes a record of all births, marriages and deaths in NSW and the date of event.

### Analysis

We analysed PBS records for 2015, the most recent calendar year for which we had complete dispensing data. Participants were excluded from analyses if they died before 1 January 2016, had missing data on study entry date, or any fields in their PBS antibiotic dispensing record were missing.

For participants with at least one linked PBS dispensing record with an ATC classification code beginning with J01 (defined as antibacterial for systemic use) [10], we estimated the number of defined daily doses dispensed per prescription by calculating the number of tablets dispensed (pack size) multiplied by their strength and dividing by the World Health Organization (WHO) Defined Daily Dose (DDD) [11]. We then calculated the quantity of antibiotics dispensed by totalling the DDDs for each prescription dispensed in 2015.

To maintain comparability with other reports [2], we converted the quantity dispensed for an individual into DDDs/1000-persons/day by dividing the total DDDs dispensed for each individual in 2015 by 365.25 and multiplying by 1000. We also examined the number of systemic antibiotic prescriptions dispensed.

We examined antibiotic use according to the following factors: age (in six categories), sex, region of residence (major cities, inner regional, outer regional/remote, unknown/missing), education (no university degree/diploma, certificate/diploma, university degree and above, unknown/missing), annual household income (<AUD20,000, AUD20,000–39,999, AUD40,000–69,999, ≥AUD70,000, unknown/missing), resident in aged care in the last year (yes, no), number of general practitioner (GP) consultations (none, 1–6, 7–9, 10–15, >15 times), and hospitalisation in the last year (yes, no) as a measure of co-morbidity. Socio-demographic variables were based on data reported at recruitment although age was calculated as that attained in 2015. Residence in aged care and number of GP consultations in the last year were ascertained by appropriate codes in the MBS (S1 Table) and hospitalisation by APDC record. To determine exposure ‘in the last year’, for antibiotic users, the index date was the date of first antibiotic dispensing in 2015; for non-users, this was the 5^th^ July 2015, the median date of antibiotic dispensing for all users in 2015.

We used Poisson regression with robust standard errors to estimate the ratio of mean DDDs/1000-persons/day by each characteristic and in the model adjusted for all characteristics examined. Such a model can be used rather than log linear regression when the outcome variable is zero [12]. Additionally, we adjusted for chronic co-morbidities including asthma and diabetes based on self-report and/or a past hospitalisation history. Finally, we conducted sensitivity analyses by repeating analyses using the number of prescriptions dispensed instead of DDDs. Analyses were conducted using STATA 14.1.

## Results

**Fig 1:**
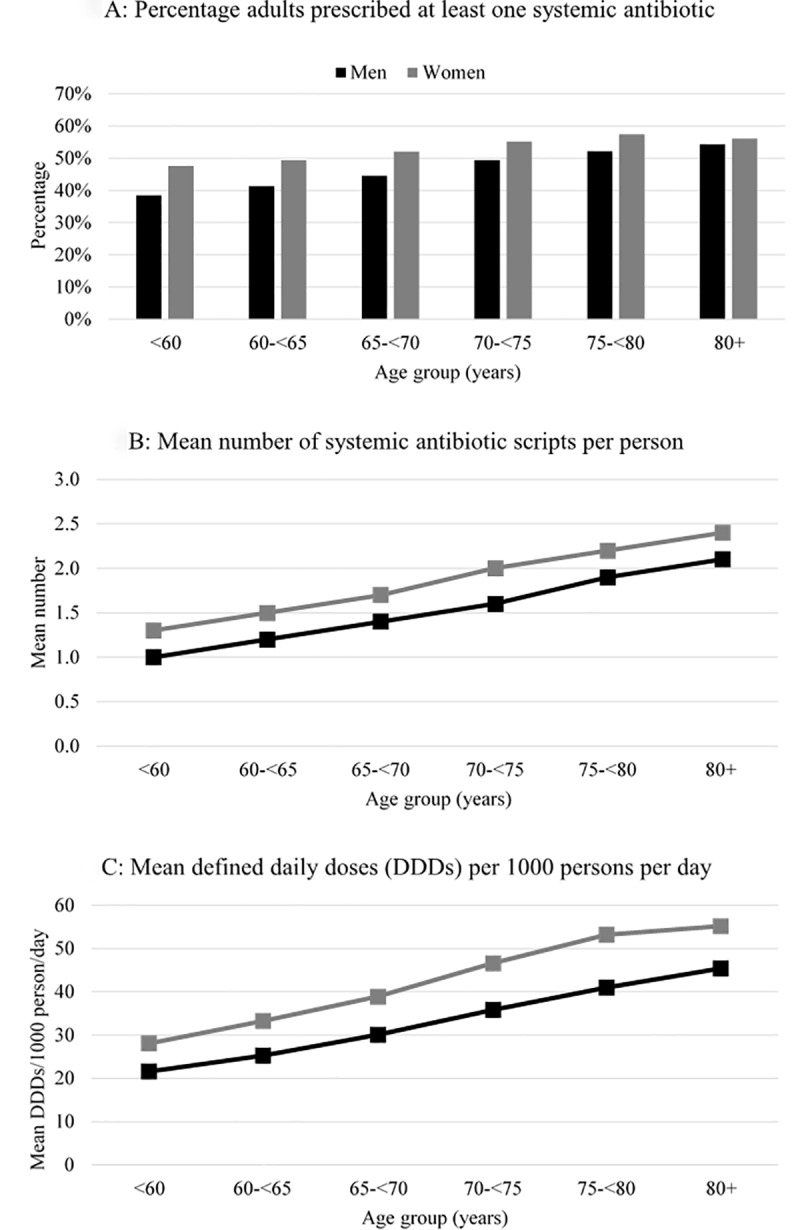
Distribution of antibiotic dispensing by age and sex in 2015.

After exclusions, there were 239,981 participants included in analyses. Participants’ median age in 2015 was 67.4 years (interquartile range: 60.5–75.6); 55.1% were women; 25.4% had an annual household income ≥AUD 70,000; 24.2% reported having a university degree or higher qualification; 51.7% were resident in a major city; 3.1% had a record indicating residence in an aged care facility in the last year; and 28.3% had a hospitalisation record in the last year. Overall, 49.3% (n = 118,271) had been dispensed at least one systemic antibiotic during 2015. The total number of antibiotic prescriptions dispensed among participants was 392,856, of which 98,298 were identified as from a repeat prescription. The mean DDDs/1000-persons/day in the cohort was 36.5. Dispensing varied with age and sex (Fig 1). The mean number of prescriptions per person and mean DDDs per day increased with increasing age, and also differed by sex with women more likely to receive antibiotics than men (Fig 1B and 1C).

Antibiotic use was highest amongst residents in aged care (80.5 DDDs/1000-persons/day) and in participants with >15 GP consultations in a year (88.3 DDDs/1000-persons/day) (Table 1). Antibiotic use also differed by age group, sex, household income, education level, and history of hospitalisation in the last year. Additionally, there was a gradient of increasing use with more GP consultations. In the multivariate model while all rate ratios were attenuated, adjusted incidence rate ratios (aIRRs) remained significantly elevated among those resident in aged care, in those with >15 GP consultations in the last year, and among those with a hospitalisation record in the last year (Table 1). Additional adjustment for a history of asthma or diabetes did not materially change findings.

**Table 1:** Quantity and incidence rate ratios of antibiotics dispensed in adults in DDDs/1000/day according to various characteristics, 2015.

	N	mean DDD/1000/day	Crude incidence rate ratio (95%CI)	Adjusted* incidence rate ratio (95%CI)
**Age group (years)**				
<60	55,830	25.6	1.00	1.00
60-<65	44,318	29.9	1.17(1.13–1.21)	1.07(1.04–1.11)
65-<70	41,555	34.9	1.37(1.32–1.41)	1.12(1.11–1.16)
70-<75	35,068	41.5	1.62(1.57–1.68)	1.17(1.12–1.20)
75-<80	25,690	47.1	1.84(1.77–1.91)	1.13(1.08–1.18)
80+	37,520	50.4	1.97(1.91–2.04)	1.06(1.01–1.10)
**Sex**				
Women	132,290	39.9	1.00	1.00
Men	107,691	32.4	0.81(0.79–0.83)	0.84(0.82–0.85)
**Annual household income (AUD)**				
lesss than 20,000	42,834	48.5	1.00	1.00
20,000–39,999	41,588	38.2	0.79(0.76–0.82)	0.94(0.91–0.97)
40,000–69,999	44,627	30.9	0.64(0.62–0.66)	0.91(0.87–0.94)
> = 70,000	61,025	26.6	0.55(0.53–0.57)	0.93(0.90–0.96)
unknown/missing	49,907	42.0	0.87(0.84–0.90)	0.97(0.94–1.01)
**Highest education level attained**				
no uni degree or diploma	127,444	40.3	1.00	1.00
Certificate or diploma	51,094	34.3	0.85(0.83–0.88)	0.99(0.96–1.02)
uni degree	57,998	29.7	0.74(0.72–0.76)	0.98(0.95–1.01)
unknown/missing	3,445	41.8	1.04(0.95–1.13)	0.95(0.87–1.03)
**Region of residence**				
cities	124,022	38.2	1.00	1.00
inner regional	84,282	35.3	0.92(0.90–0.95)	0.96(0.94–0.97)
outer regional/remote	27,129	33.5	0.88(0.85–0.91)	0.92(0.89–0.95)
unknown/missing	4,548	32.0	0.84(0.78–0.91)	0.96(0.89–1.03)
**Resident in aged care in last year**				
no	232,871	35.2	1.00	1.00
yes	7,110	80.5	2.29(2.18–2.41)	1.27(1.20–1.34)
**No. of GP visits in last year**				
0	13,709	5.2	0.26 (0.23–0.29)	0.26(0.24–0.30)
1 to 6	112,522	20.2	1.00	1.00
7 to 9	43,094	38.0	1.88(1.83–1.93)	1.76(1.71–1.81)
10 to 15	42,522	54.0	2.67 (2.60–2.74)	2.40(2.33–2.47)
>15	28,134	88.3	4.37(4.25–4.50)	3.68(3.56–3.81)
**Hospitalisation in last year**				
no	172,847	29.6	1.00	1.00
yes	67,134	54.2	1.83(1.79–1.87)	1.29(1.28–1.34)

*adjusted for all characteristics in the table

**Fig 2:**
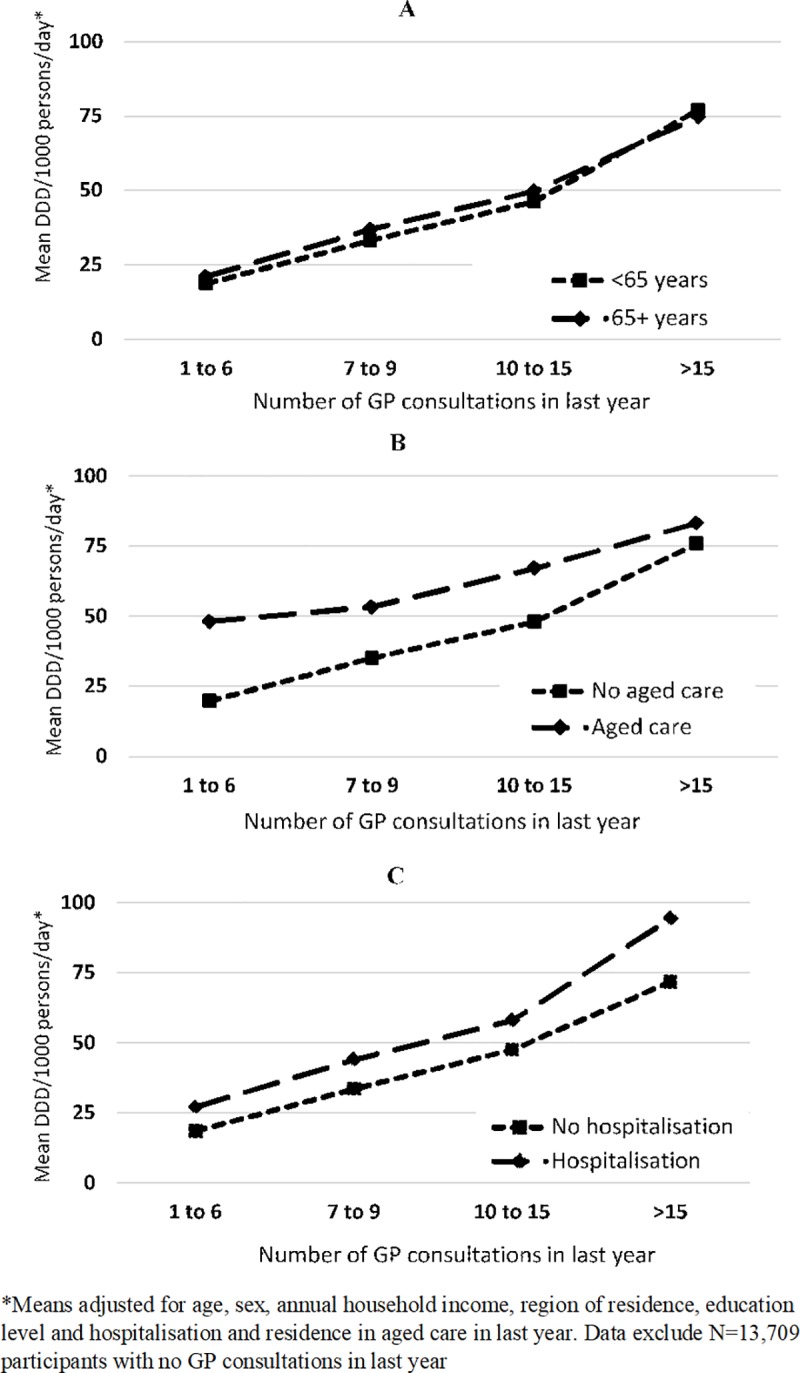
Mean DDD of systemic antibiotics dispensed per 1000 persons per day according to number of GP consultations and age (A), residence in aged care in the last year (B) and hospitalisations in last year (C).

The adjusted mean DDDs/1000-persons/day of antibiotics dispensed did not differ substantially by age strata once the number of GP consultations in the last year was considered (Fig 2A). Among those consulting GPs >15 times, the adjusted mean antibiotic DDDs/1000-persons/day was 77.1 and 74.8 in those aged <65 and those 65+ years, respectively. When stratified by residence in aged care, the pattern of antibiotic use according to GP visits did differ (Fig 2B), with those in aged care having greater antibiotic use although differences were less pronounced in those with >15 GP consultations. Antibiotic use according to number of GP consultations was consistently greater in those who had been hospitalised in the last year compared to those not (Fig 2C).

Sensitivity analyses comparing mean prescription counts were consistent with analyses of DDDs. The highest number of antibiotic prescriptions dispensed was among residents in aged care (mean 3.7 prescriptions/person) and in participants who had >15 GP consultations a year (mean 3.9 prescriptions/person). The adjusted rate ratios were similar to that for DDDs (S2 Table).

## Discussion

In this large population-based study of older Australians, we quantified the use of antibiotics in the community at an individual-level and estimated differences in use according to socio-demographic characteristics and admission to aged care facilities and hospitals. While earlier Australian reports using dispensing data indicate that increasing age is a major factor associated with the increased use of antibiotics among adults [13], they have not been able to examine how this relationship is influenced by other factors associated with aging such as residence in aged care or co-morbidity. We found that while average use of antibiotics increased with increasing age, factors such as the frequency of visiting a GP and residence in aged care may be more important. After accounting for age and other factors, those in aged care had about 30% greater use of antibiotics than those not in aged care. Those visiting GPs >15 times/year used almost four-times the amount of antibiotics as those visiting GPs 1–6 times/year. Those hospitalised in the last year had 30% greater use of antibiotics, whilst use was 16% higher comparing women to men and 7% higher comparing low income to high income individuals.

Previous studies have reported mixed findings regarding what factors are associated with greater antibiotic use in the community or outpatient setting. A recent systematic review [14] examining antibiotic prescribing for respiratory tract infections found 10 out of 19 studies suggested increasing age was associated with greater antibiotic use but that there were no consistent relationships with sex, geographic location, and presence of co-morbidities. The studies included in this systematic review were primarily from the US, included a variety of age ranges, and had sample sizes ranging from 300–4800 individual patients although two studies had samples of >100,000 patient encounters. In contrast, a recent US study of 260,000 patient encounters where 21% were for presumed viral respiratory tract infections reported that antibiotic prescribing was associated with shorter consultation time, female sex, private insurance status, seeing a family doctor (compared to specialists), and physician prescribing habit [15].

In Australia, an analysis of the Australia-wide MedicineInsight database, which contains records from over 3 million patients of all ages attending GPs around Australia, reported about 30% of patients were prescribed antibiotics in 2015 [2]. They also described higher prescribing among women, older patients, and those living in cities [2]. Another Australian report limited their analyses to antibiotic prescribing for upper respiratory tract infections and included both hospital and community-based prescribing [16]. While this study reported greater likelihood of antibiotic prescribing in hospitals and in upper respiratory tract infections with more localised symptoms and signs, they reported no significant differences in prescribing by patient age, gender, rurality, and Indigenous status. However, their sample size was small (N = 698) perhaps limiting the ability to detect such differences.

Our findings are based on a very large sample of Australian adults, actual antibiotic dispensing data rather than prescriptions, prospectively collected socio-demographic information, and independently collected data on recent GP visits, residence in aged care and hospitalisation. Aged care settings are increasingly a focus of antimicrobial stewardship activities as high use of antibiotics have been reported in earlier surveys [17]. Our data add to the aged care surveys by quantifying differences in use between those in aged care and community-based populations and, uniquely, adjusts estimates of use for differences in age, sex and co-morbidities. The strong association between antibiotic use with increasing GP visits has not been reported previously in Australian studies. This finding seems intuitive as most community antibiotic prescribing would be from GPs and it is likely that those visiting GPs more often have greater health needs. However it is possible that more GP visits may also reflect shorter consultations, a factor found in the US study [15] to be associated with greater antibiotic prescribing. The very substantial use of antibiotics in adults having many GP visits deserves greater investigation to better understand what may be contributing to this.

Due to differences in study populations (e.g. age), and methods of ascertainment of antibiotic use (dispensing rather than prescribing), our findings may not be directly comparable to other studies. Data for the whole Australian population derived from PBS dispensing data found that 44.7% of the Australian population had at least one antibiotic dispensed in 2015 with an average of 25.4 DDDs/1000/day [2]. The age-specific proportions of those dispensed antibiotics in our cohort were a few percentage points lower than that reported in this previous analysis, particularly among those aged >65 years. This may reflect the fact that participants in the 45 and Up cohort study may be healthier than the general population of a similar age, although it is less likely to affect the comparisons of use (rate ratios) between groups [18].

Other potential limitations of our data include the lack of data on private prescriptions as these are not included in the PBS data. An earlier study estimated about 5% of all systemic antibiotics would be supplied on private prescription [19]. It is possible this may account for the small differential in dispensing according to household income that we observed. Also, ascertainment of residence in aged care was based on MBS item numbers for services dispensed in an aged care facility (S1 Table). As many residents in aged care facilities may still be able to attend their healthcare provider, this might result in under-ascertainment of residence in aged care in our analyses, leading to potential dilution of any differences observed. Finally, we excluded participants who died in 2015 as we wanted to include equivalent observation time for all study participants. This could result in an underestimate of the total antibiotic dispensing, particularly in the older age groups. This could also explain the differences in the estimate of the proportion of the cohort dispensed antibiotics between the whole-of-Australia data [2] and our cohort.

## Conclusions

In summary, we found that residence in aged care and high number of GP visits were associated with substantially greater antibiotic use even after adjusting for age, sex, socio-demographic characteristics and comorbidity. Future analyses examining antibiotic classes in these high utilisation subgroups are needed to better understand the patterns and factors contributing to the differences observed. Our study also provides important evidence regarding high use groups to inform antimicrobial stewardship.

## Supporting information

S1 Table MBS codes used for GP consultations and GP consultations in aged care facilities. (DOCX)

S2 Table Sensitivity analysis of number of systemic antibiotic prescriptions dispensed and incidence rate ratios in adults according to various characteristics, 2015. (DOCX)
