# Supplementary material for: Socio-demographic and health service factors associated with antibiotic dispensing in older Australian adults
Source: PLoS One. 2019 Aug 29;14(8):e0221480. doi: 10.1371/journal.pone.0221480 (PMC6715220; doi:10.1371/journal.pone.0221480)
Supplement: S1 Table — (DOCX) [file pone.0221480.s001.docx]

Supplementary Table 1: MBS codes used for GP consultations and GP consultations in aged care facilities

| **GP consultations** | |
| --- | --- |
| MBS item number | Description |
| Items 3 to 51 | Attendances by General Practitioners |
| Items 193,195,197,199, 597, 599 | Attendances by General Practitioners |
| Items 2497 to 2559 | Attendances by General Practitioners |
| Items 5000-5067 | Attendances by General Practitioners |
| **Participants aged care facility attendance** | |
| MBS item number | Description |
| Items 20, 35, 43, 51, 92, 93, 95, 96, 5010, 5028, 5049, 5067, 5260, 5263, 5265, 5267 | Residential Aged Care Facility Attendances |
| Item 731 | Contribution to a Multidisciplinary Care Plan, or to a review of a multidisciplinary care plan, for a resident in an aged care facility |
| Item 903 | Residential Medication Management Review |
| Items 2125, 2138, 2179, 2220 | Medical practitioner telehealth attendances at a residential aged care facility |
| Items 10947, 10948, | At the time of the attendance, is located at a residential aged care facility |
| Items 73934, 73935, | Approved pathology authority from in a residential aged care home or institution |
| Item 10984 | A care recipient receiving care in a residential aged care service |
| Items 82223, 82224, 82225 | Telehealth attendance at a residential aged care facility |
