# Supplementary material for: Socio-demographic and health service factors associated with antibiotic dispensing in older Australian adults
Source: PLoS One. 2019 Aug 29;14(8):e0221480. doi: 10.1371/journal.pone.0221480 (PMC6715220; doi:10.1371/journal.pone.0221480)
Supplement: S2 Table — (DOCX) [file pone.0221480.s002.docx]

Supplementary Table 2: Sensitivity analysis of number of systemic antibiotic prescriptions dispensed and incidence rate ratios in adults according to various characteristics, 2015

|  | N | mean number of prescriptions dispensed | Crude incidence rate ratio (95%CI) | Adjusted* incidence rate ratio (95%CI) |
| --- | --- | --- | --- | --- |
| **Age group (years)** |  |  |  |  |
| <60 | 55,830 | 1.2 | 1.00 | 1.00 |
| 60-<65 | 44,318 | 1.4 | 1.14(1.12-1.17) | 1.05(1.02-1.07) |
| 65-<70 | 41,555 | 1.6 | 1.33(1.30-1.36) | 1.09(1.07-1.12) |
| 70-<75 | 35,068 | 1.8 | 1.55(1.51-1.58) | 1.10(1.08-1.13) |
| 75-<80 | 25,690 | 2.1 | 1.74(1.70-1.79) | 1.07(1.04-1.10) |
| 80+ | 37,520 | 2.3 | 1.90(1.88-1.95) | 1.02(0.99-1.04) |
| **Sex** |  |  |  |  |
| Women | 132,290 | 1.8 | 1.00 | 1.00 |
| Men | 107,691 | 1.5 | 0.84(0.83-0.86) | 0.87(0.86-0.88) |
| **Annual household income (AUD)** | |  |  |  |
| <20,000 | 42,834 | 2.2 | 1.00 | 1.00 |
| 20,000-39,999 | 41,588 | 1.7 | 0.79(0.77-0.81) | 0.94(0.92-0.96) |
| 40,000-69,999 | 44,627 | 1.4 | 0.65(0.64-0.67) | 0.92(0.90-0.94) |
| >=70,000 | 61,025 | 1.2 | 0.56(0.55-0.57) | 0.94(0.91-0.96) |
| unknown/missing | 49,907 | 1.9 | 0.87(0.85-0.90) | 0.98(0.95-1.00) |
| **Highest education level attained** | |  |  |  |
| no university degree/diploma | 127,444 | 1.8 | 1.00 | 1.00 |
| Certificate or diploma | 51,094 | 1.5 | 0.85(0.84-0.87) | 0.98(0.97-1.00) |
| university degree | 57,998 | 1.3 | 0.73(0.72-0.75) | 0.96(0.94-0.98) |
| unknown/missing | 3,445 | 1.9 | 1.05(0.99-1.11) | 0.96(0.91-1.01) |
| **Region of residence** |  |  |  |  |
| cities | 124,022 | 1.7 | 1.00 | 1.00 |
| inner regional | 84,282 | 1.6 | 0.90(0.89-0.92) | 0.95(0.93-0.96) |
| outer regional/remote | 27,129 | 1.5 | 0.89(0.87-0.92) | 0.94(0.92-0.96) |
| unknown/missing | 4,548 | 1.5 | 0.87(0.82-0.92) | 0.99(0.94-1.04) |
| **Resident in aged care in last year** | |  |  |  |
| no | 232,871 | 1.6 | 1.00 | 1.00 |
| yes | 7,110 | 3.7 | 2.32(2.24-2.40) | 1.31(1.26-1.35) |
| **No of GP visits in last year** |  |  |  |  |
| 0 | 13,709 | 0.2 | 0.24(0.22-0.26) | 0.24(0.23-0.27) |
| 1 to 6 | 112,522 | 0.9 | 1.00 | 1.00 |
| 7 to 9 | 43,094 | 1.7 | 1.85(1.81-1.88) | 1.75(1.71-1.78) |
| 10 to 15 | 42,522 | 2.4 | 2.61(2.57-2.67) | 2.38(2.34-2.43) |
| >15 | 28,134 | 3.9 | 4.27(4.19-4.35) | 3.67(0.59-3.75) |
| **Hospitalisation in last year** |  |  |  |  |
| no | 172,847 | 1.4 | 1.00 | 1.00 |
| yes | 67,134 | 2.4 | 1.77(1.75-1.80) | 1.28(1.25-1.30) |

*incidence rate ratio adjusted for age, sex, residence, income, education, aged care, number of GP visits and hospitalisation
